# A randomised, controlled crossover comparison of the C-MAC videolaryngoscope with direct laryngoscopy in 150 patients during routine induction of anaesthesia

**DOI:** 10.1186/1471-2253-11-6

**Published:** 2011-03-01

**Authors:** Erol Cavus, Carsten Thee, Thora Moeller, Joerg Kieckhaefer, Volker Doerges, Klaus Wagner

**Affiliations:** 1 Consultant in Anaesthesiology, Department of Anaesthesiology and Intensive Care Medicine, University Hospital Schleswig-Holstein, Campus Kiel, Schwanenweg 21, 24105 Kiel, Germany; 2 Resident, Department of Anaesthesiology and Intensive Care Medicine, University Hospital Schleswig-Holstein, Campus Kiel, Schwanenweg 21, 24105 Kiel, Germany; 3 Consultant in Anaesthesiology, Department of Anaesthesiology and Intensive Care Medicine, Suedstadt Hospital Rostock, Suedring 81, 18059 Rostock, Germany; 4 Professor of Anaesthesiology, Department of Anaesthesiology and Intensive Care Medicine, University Hospital Schleswig-Holstein, Campus Kiel, Schwanenweg 21, 24105 Kiel, Germany; 5 Associate Professor of Anaesthesiology and chair, Department of Anaesthesiology and Intensive Care Medicine, Suedstadt Hospital Rostock, Suedring 81, 18059 Rostock, Germany

## Abstract

**Background:**

The C-MAC^® ^(Karl Storz, Tuttlingen, Germany) has recently been introduced as a new device for videolaryngoscopy guided intubation. The purpose of the present study was to compare for the first time the C-MAC with conventional direct laryngoscopy in 150 patients during routine induction of anaesthesia.

**Methods:**

After approval of the institutional review board and written informed consent, 150 patients (ASA I-III) with general anaesthesia were enrolled. Computer-based open crossover randomisation was used to determine the sequence of the three laryngoscopies: Conventional direct laryngoscopy (HEINE Macintosh classic, Herrsching, Germany; blade sizes 3 or 4; *DL *group), C-MAC size 3 (*C-MAC3 *group) and C-MAC size 4 (*C-MAC4 *group) videolaryngoscopy, respectively. After 50 patients, laryngoscopy technique in the C-MAC4 group was changed to the straight blade technique described by Miller (C-MAC4/SBT).

**Results:**

Including all 150 patients (70 male, aged (median [range]) 53 [20-82] years, 80 [48-179] kg), there was no difference of glottic view between DL, C-MAC3, C-MAC4, and C-MAC4/SBT groups; however, worst glottic view (C/L 4) was only seen with DL, but not with C-MAC videolaryngoscopy. In the subgroup of patients that had suboptimal glottic view with DL (C/L≥2a; n = 24), glottic view was improved in the C-MAC4/SBT group; C/L class improved by three classes in 5 patients, by two classes in 2 patients, by one class in 8 patients, remained unchanged in 8 patients, or decreased by two classes in 1 patient. The median (range) time taken for tracheal intubation in the DL, C-MAC3, C-MAC4 and C-MAC4/SBT groups was 8 sec (2-91 sec; n = 44), 10 sec (2-60 sec; n = 37), 8 sec (5-80 sec; n = 18) and 12 sec (2-70 sec; n = 51), respectively.

**Conclusions:**

Combining the benefits of conventional direct laryngoscopy and videolaryngoscopy in one device, the C-MAC may serve as a standard intubation device for both routine airway management and educational purposes. However, in patients with suboptimal glottic view (C/L≥2a), the C-MAC size 4 with straight blade technique may reduce the number of C/L 3 or C/L 4 views, and therefore facilitate intubation. Further studies on patients with difficult airway should be performed to confirm these findings.

## Background

Since poor glottic visualisation is encountered between 1-9% of intubation attempts [1,2], in recent years the technique of videolaryngoscopy has begun to play an important role in the management of patients with an unanticipated difficult or failed laryngoscopic intubation [3]. More recently, the portable C-MAC^® ^videolaryngoscope (Karl Storz, Tuttlingen, Germany), a further development of previous videolaryngoscopes by Karl Storz (MVL, V-MAC), has been introduced into clinical practice; we have shown previously in a preliminary clinical study that the C-MAC may be a useful alternative in both routine and difficult airway management, and may additionally be used for educational purposes [4]. A study performed on manikins has shown advantages of the C-MAC over both the standard Macintosh laryngoscopes and other indirect laryngoscopes [5].

The present clinical study was designed to compare for the first time the use of the C-MAC videolaryngoscope with conventional direct laryngoscopy (Macintosh) in 150 patients with both normal and difficult airways during routine induction of anaesthesia. Primary endpoint was change of glottic visualisation; secondary endpoints were time to tracheal intubation and success rate.

## Methods

**Figure 1:**
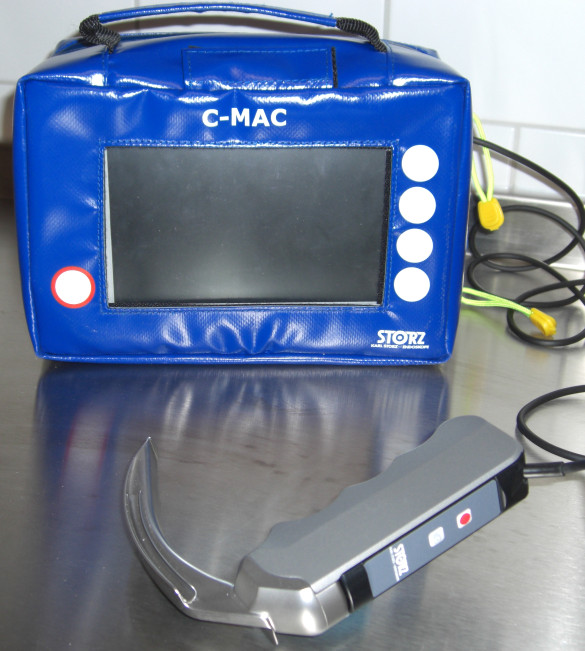
**The portable C-MAC videolaryngoscope**. The C-MAC videolaryngoscope, stored in the portable protective bag. Note the buttons for image recording on the monitor and the laryngoscope handle.

**Figure 2:**
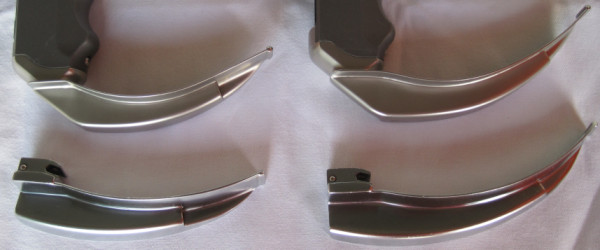
**Comparison of C-MAC videolaryngoscopes with Macintosh laryngoscopes**. Blade shapes of C-MAC videolaryngoscopes (top) and conventional Macintosh laryngoscopes (bottom) sizes 3 and 4, respectively.

We have described the C-MAC^® ^videolaryngoscope (Karl Storz, Tuttlingen, Germany; figure 1) in detail elsewhere [4]. Briefly, compared to the MVL as described by Kaplan et al. [6], this stainless steel blade retains the original Macintosh shape. It has a closed blade design with no edges and gaps for hygienic traps, and is, so far, available in three sizes (2, 3 and 4). The C-MAC blade provides a slim profile (max. 14 mm), and the edges are slanted to avoid damage to the mouth and teeth. Further, the C-MAC incorporates Lithium-Ion battery technology with at least two hours capacity, the smallest possible (2 mm) digital camera (CMOS, 320 × 240 pixels), and a high power LED. The view obtained includes the tip of the blade and, therefore, allowing for guiding the tip of the blade into the vallecula under vision. In contrast to other videolaryngoscopes, due to the original Macintosh blade shape (figure 2), conventional direct view of the glottis is also available. Like other videolaryngoscopes, a view of the epiglottis and glottis is available on the video screen as soon as the camera section of the C-MAC enters the pharynx.

After approval of the institutional review board (University Kiel, Schleswig-Holstein, Germany) and obtaining written informed consent, 150 patients (ASA I-III) of either gender, undergoing elective surgery in the supine position between August 2009 and November 2009 with general anaesthesia in whom tracheal intubation was indicated, were enrolled in the study. Computer-based open crossover randomisation was used to determine the sequence of the three laryngoscopies: Conventional direct laryngoscopy (HEINE Macintosh classic, Herrsching, Germany; blade sizes 3 or 4; *DL *group), C-MAC size 3 (*C-MAC3 *group) and C-MAC size 4 (*C-MAC4 *group) videolaryngoscopy, respectively. After 50 patients, videolaryngoscopic technique in the C-MAC4 group was changed to the straight blade technique described by Miller [7,8] (*C-MAC4 *changed to *C-MAC4/SBT *group).

Patients were excluded if pathology of the upper respiratory or alimentary tract were known or suspected or if a rapid sequence induction was indicated. In addition, patients were excluded if an awake intubation was appropriate due to a suspected or known difficult airway. Existence of predictors for difficult direct laryngoscopy alone was no exclusion criteria. Preoperatively we scored the cervical extension of the head (atlanto-occipital extension according to Bellhouse [9]), the thyromental distance described by Patil [10], the inter-incisor distance and the view of the oropharynx on mouth-opening described by Mallampati [11], and modified by Samsoon and Young [12].

Standard monitoring devices were attached before induction of anaesthesia, including non-invasive arterial blood pressure, heart rate (HR), and oxygen saturation (SpO_2_; S/5, Datex-Ohmeda, Helsinki, Finland). The patient's head was supported on a firm pillow with an appropriate height to achieve a sniffing position. After 3 min preoxygenation with a face mask, anaesthesia was induced with remifentanil 0.3 μg kg^-1 ^min^-1 ^and propofol 1.5-2.5 mg kg^-1^. Appropriate neuromuscular blockade was produced by rocuronium 0.6 mg kg^-1^, and was confirmed using a peripheral nerve stimulator (train-of-four count = 0) before airway manipulation.

Next, all patients underwent three separate laryngoscopies using the standard Macintosh laryngoscope with an appropriate size 3 or 4, the C-MAC size 3, and the C-MAC size 4 videolaryngoscope, respectively, in the sequence determined by randomisation. The blade was introduced to the right of the tongue and advanced toward the vallecula by one of three anaesthesiologists with at least eight years experience (after being trained on manikins with the C-MAC). The position of the device was adjusted to have both the glottis and the blade-tip in the centre of either view or screen, and tracheal intubation was performed with the last blade used. The attending anaesthesiologist was requested to identify the best achievable Cormack-Lehane (C/L) view [13], modified by Yentis-Lee [14], with direct laryngoscopy (conventional Macintosh laryngoscope) and videolaryngoscopy (C-MAC videolaryngoscope); depending on necessity, the use of external laryngeal manipulation (BURP manoeuvre [15]) at the discretion of the anaesthesiologist was allowed, but not prescribed. All Cormack-Lehane (C/L) classification data with the C-MAC videolaryngoscope applied to the video view seen on the monitor. As usual for Macintosh-shaped blades, tube insertion was performed from the right side, and no kind of stylets was used a priori. If it was necessary to give the endotracheal tube a specific shape to guide the tube to the glottic entrance, a semi-flexible tube-guide (Flexislip, Rüsch, Teleflex Medical Europe, Ireland), whose tip remained in the tube, was used. Further, no anti-fogging substances were used at all. We recorded the ease or difficulty of intubation with each device, the time to optimal laryngoscopic view (time from touching the device to optimal view) and the time for endotracheal intubation (time from touching the tube to successful endotracheal placement). Further, the number of intubation attempts was recorded; every time the tube was newly advanced to the glottic entrance was recorded as a new attempt. Upon completion of the study protocol, the anaesthesiologist gave a subjective assessment of handling, which was rated as very good, good, and poor, and of common concerns (comfort, guidance of laryngoscope handle, blade insertion, and glottic exposure). Correct tube position, and subsequently successful ventilation was assessed with capnography and bilateral chest auscultation. Peripheral oxygen saturation and heart rate were recorded continuously, and mean arterial blood pressure was recorded intermittently every five minutes.

### Statistical analysis

Based on data of a preliminary investigation [4], we calculated the sample size to detect at least a difference of one class between devices in the primary end-point glottic visualisation (C/L) with a type I-error of 0.05 and a power of 0.9; required sample size was 97. Data are expressed as median [range], mean ± standard deviation (SD), or absolute numbers (percentage), as appropriate. Kruskal-Wallis one-way ANOVA with Dunn's post-hoc test was used to evaluate differences among the groups in the dependent parameters of intubation time, attempts, and overall satisfaction for the respective laryngoscopy groups, and in the C/L grades. Statistical significance was considered at *p <*0.05.

## Results

Patients' characteristics are shown in table 1. All patients showed stable haemodynamic conditions before, during, and after laryngoscopy. Peripheral oxygen saturation was maintained above 95% in all patients throughout the laryngoscopy and intubation period. We did not detect any injury of the palatoglossal arch or dental injury in any of the patients.

**Table 1:** Patient characteristics in the DL, C-MAC3, C-MAC4, and C-MAC/STB groups according to intubating device used.

	DL	C-MAC3	C-MAC4	C-MAC/STB
Number intubated N =	50	37	18	45
Age (years)	49 (23-82)	54 (20-74)	46 (34-72)	58 (27-79)
Sex (female/male)	29/21	27/10	7/11	17/28
Body weight (kg)	81 (60-179)	76 (54-98)	82 (54-150)	78 (48-135)
Height (cm)	170 (156-196)	168 (150-186)	173 (163-188)	173 (155-193)
Body mass index (BMI)	27 (20-63)	27 (20-40)	27 (20-41)	27 (19-44)
Mallampati class (I/II/III/IV)	16/20/13/1	8/23/6/0	4/6/7/1	9/21/15/0
Inter-incisor distance (cm)	5 ± 1	5 ± 1	5 ± 1	5 ± 1
Thyromental distance (Patil; cm)	8 ± 2	7 ± 4	8 ± 3	8 ± 3
Cervical extension (Bellhouse;</> 15°)	3/47	2/35	2/16	5/40

Data are given as median (range), mean ± SD, or numbers.

**Figure 3:**
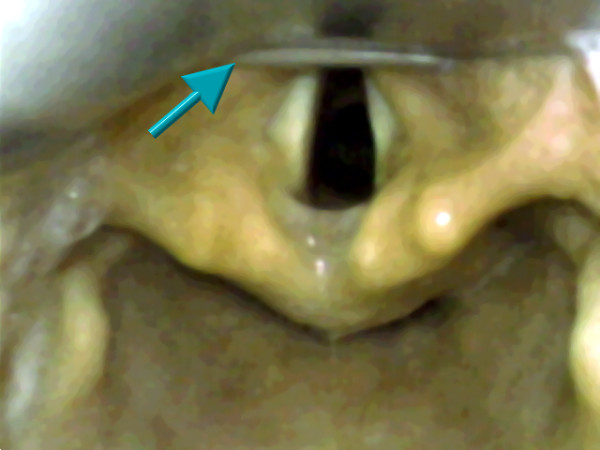
**Image capture of C-MAC laryngoscopic view**. Image capture of videolaryngoscopic view with a C-MAC blade size 4 (formerly Cormack-Lehane class 3 view changed to class 1 view). Note the epiglottis that is uploaded on the blade tip (arrow; C-MAC4/SBT group).

Since it became obvious after 50 patients that videolaryngoscopy with the C-MAC size 4 blade provided no benefit over videolaryngoscopy with the C-MAC size 3 blade when using Macintosh laryngoscopic technique, we changed laryngoscopic technique in the C-MAC4 group to the straight blade technique (C-MAC4/SBT; figure 3).

**Figure 4:**
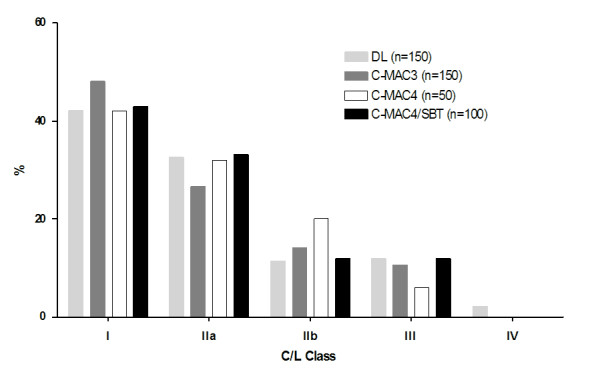
**Comparison of glottic visualisation between direct laryngoscopy and C-MAC videolaryngoscopy**. Glottic visualisation according to Cormack-Lehane score after direct Macintosh laryngoscopy and C-MAC videolaryngoscopic views in the DL, C-MAC3, C-MAC4, and C-MAC4/SBT groups, respectively. Data are given as percentage. *C/L *indicates Cormack and Lehane view,[13] modified by Yentis and Lee;[14]*DL*: Direct laryngoscopy.

**Figure 5:**
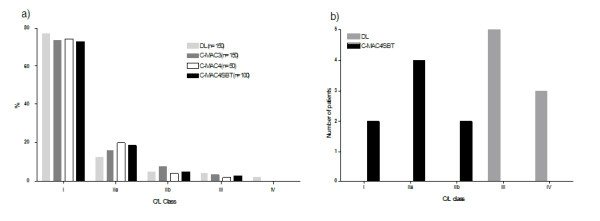
**Glottic visualisation according to Cormack-Lehane score with direct laryngoscopy and C-MAC videolaryngoscopy**. a) Best achievable Cormack-Lehane score (with external laryngeal manoeuvres, e.g. BURP) after direct laryngoscopy and C-MAC views in the DL, C-MAC3, C-MAC4, and C-MAC4/SBT groups, respectively. Data are given as percentage. *C/L *indicates Cormack and Lehane view,[13] modified by Yentis and Lee;[14]*DL*: Direct laryngoscopy. b) Improvement of Cormack-Lehane score in all patients with limited direct laryngoscopic view (DL group: C/L 3+4) after laryngoscopy with the C-MAC4/SBT (n = 8). Data are given as absolute numbers. *C/L *indicates Cormack and Lehane view,[13] modified by Yentis and Lee;[14]*DL*: Direct laryngoscopy.

The median (range; mean ± SD) time from touching the laryngoscope to optimal laryngoscopic view was 7 sec (3-45 sec; 8 ± 4 sec) in the DL group, 8 sec (2-53 sec; 11 ± 12 sec) in the C-MAC3 Macintosh group, 9 sec (4-58 sec; 18 ± 14 sec) in the C-MAC4 Macintosh group, and 8 sec (2-70 sec; 12 ± 12 sec) in the C-MAC4/SBT group (overall p = 0.21, ns), respectively. A comparable initial glottic view according to C/L score in DL, C-MAC3 Macintosh, C-MAC4 Macintosh, and C-MAC4/SBT groups is shown in figure 4. C/L view after extralaryngeal manipulation, such as BURP manoeuvre, could be improved and resulted in a glottic view as shown in figure 5a; a C/L 4 glottic view was only seen with DL, but not with C-MAC videolaryngoscopy (figure 5b). Twenty patients had better C/L classes with any C-MAC compared to DL, and 6 patients had better view with DL compared to C-MAC. In the subgroup of patients that had suboptimal glottic view ((C/L≥2a); n = 24) with DL, glottic view was improved in the C-MAC4/SBT group (table 2); C/L class improved by three classes in 5 patients, by two classes in 2 patients, by one class in 8 patients or remained unchanged in 8 patients. In one patient of the C-MAC4/SBT group, C/L grade was reduced by two classes compared to DL.

**Table 2:** Comparison of direct laryngoscopy (DL) and C-MAC4 with Miller technique (C-MAC4/SBT) views in patients with sub-optimal conventional laryngoscopic view (C/L≥2a).

	C-MAC4/SBT views				
	
DL views	C/L I	C/L IIa	C/L IIb	C/L III	C/L IV
C/L IIa, *n = 11*	5	5	1	0	0
C/L IIb, *n = 5*	1	2	2	0	0
C/L III, *n = 5*	2	1	1	1	0
C/L IV, *n = 3*	0	3	0	0	0
*Totals n = 24*	*8*	*11*	*4*	*1*	*0*

Data are given as absolute numbers. *C/L *indicates Cormack and Lehane view,[13] modified by Yentis and Lee;[14]*DL*: Direct laryngoscopy.

Successful intubation was performed in median (range; mean ± SD) after 8 sec (2-91 sec; 11 ± 14 sec) in the DL group (n = 50), 10 sec (2-60 sec; 16 ± 16 sec) in the C-MAC3 Macintosh group (n = 37), 8 sec (5-80 sec; 21 ± 24 sec) in the C-MAC4 Macintosh group (n = 18), and 11 sec (2-70 sec; 15 ± 13 sec) in the C-MAC4/SBT group (n = 45), respectively (overall p = 0.32, ns). As a trend, videolaryngoscopy resulted in more intubation attempts than DL (table 3); however, in 6 patients with limited glottic visualisation (C/L≥ 3) that were originally ought to be intubated by DL, intubation had to be performed with the C-MAC4 and straight blade technique (figure 3). Taking this into account, intubation success rates with DL, C-MAC3, C-MAC4, and C-MAC4/STB were 44/50 (88%), 37/37 (100%), 18/18 (100%), and 51/51 (100%), respectively. Use of a semi-flexible tube-guide to guide the endotracheal tube to the visualised glottis was mandatory in 1 (DL), 2 (C-MAC3), 4 (C-MAC4), and 6 (C-MAC4/SBT) patients, respectively. Out of 150 patients, fogging of the optical lens was observed transiently in 11 cases, and the monitor's view was insufficient in 2 cases due to dazzling.

**Table 3:** Number of intubation attempts and subjective assessment of handling with direct laryngoscopy (DL), C-MAC3, C-MAC4, and C-MAC4/SBT videolaryngoscopy.

	DL	C-MAC3	C-MAC4	C-MAC4/SBT
	(n = 150)	(n = 150)	(n = 50)	(n = 100)
Number of intubation attempts				
1	48/50 (96%)	27/37 (73%)	14/18 (78%)	33/45 (73%)
2	2/50 (4%)	10/37 (27%)	3/18 (17%)	8/45 (18%)
3	0*	0	1/18 (5%)	4/45 (9%)
Subjective assessment of handling (C/L < 3 with DL)				
very good	85 (57%)	62 (41%)	12 (24%)	39 (39%)
good	49 (33%)	63 (42%)	34 (68%)	39 (39%)
poor	7 (5%)	16 (11%)	3 (6%)	14 (14%)
Subjective assessment of handling (C/L≥3 with DL)				
very good	0 (0%)	2 (1%)	0 (0%)	2 (2%)
good	5 (3%)	4 (2%)	1 (2%)	4 (4%)
poor	4 (2%)	3 (2%)	0 (0%)	2 (2%)
Common concerns				
comfort	6 (4%)	16 (11%)	2 (4%)	16 (16%)
guidance of laryngoscope handle	6 (4%)	13 (9%)	3 (6%)	14 (14%)
blade insertion	8 (5%)	12 (8%)	5 (10%)	9 (9%)
glottic exposure	26 (17%)	27 (18%)	8 (16%)	21 (21%)

Data are given as absolute numbers (percentage). *DL*: Direct laryngoscopy. *Six patients that were originally ought to be intubated by DL showed limited glottic visualisation (C/L≥ 3) and had to be intubated with the C-MAC4 and straight blade technique.

## Discussion

The present study shows that 1) the use of the C-MAC videolaryngoscope provides comparable or better glottic views than direct laryngoscopy, and 2) in patients with impeded glottic view (C/L≥2a), C/L class may be improved and subsequently patients may be intubated with the C-MAC4 blade combined with the use of Miller's "straight blade technique".

The C-MAC videolaryngoscope is a relatively new device with the unique advantage that it provides the possibility to obtain both a direct laryngoscopic view and a camera view that is displayed on the video screen, in contrast to many previous videolaryngoscopes.^a ^On the one hand, this may be very helpful for educational purposes, since the student is enabled to follow an ideal intubation process on the video screen, and thereafter, the instructor may directly observe the student's intubation attempts. On the other hand, this may have important ramifications, if the video view is worse than the direct view, as observed with the C-MAC in six patients of the present study, or the intubation itself is difficult due to a high blade angulation, as shown with the GlideScope [16]. The user therefore is in the comfortable situation to decide with a single device whether to intubate by direct laryngoscopic or videolaryngoscopic view, depending on the better view provided, which has been addressed previously [4]. Lower angulations of the blade, times for laryngoscopy and intubation comparable to direct laryngoscopy as well as good handling conditions in the present study suggest that the C-MAC size 3 may be the standard device to use in daily practice; analogous to the standard Macintosh laryngoscope, the C-MAC size 4 may be used in larger patients. In our opinion, in all cases with easy intubation conditions the anaesthesiologist should prefer the direct laryngoscopic view of the C-MAC 3 over the videolaryngoscopic view. However, this issue may be debatable, since increased forces on the maxillary incisors with conventional laryngoscopy compared to videolaryngoscopy have been observed during difficult intubation [17], but more importantly, videolaryngoscopy-guided intubation has the potential risk of increasing the number of intubation attempts and time, and the use of a tube-guide, respectively, as shown in the present study.

Similar to experiences from previous Storz videolaryngoscopes [18,19], an endotracheal tube stylet or semi-flexible tube-guide is not mandatory due to the original Macintosh shape of the blade: only 12 of 150 patients in the present study, including 6 of 8 patients with highly limited direct laryngoscopic view, were intubated with the help of a tube-guide. This finding is even more significant because we did not exclude patients with morbid obesity. Even if the safety of using or not using a stylet or tube-guide may be debatable, there have been reports of complications such as oropharyngeal perforations with the use of a previous videolaryngoscope (GlideScope) [20-24]; in that device, a highly angulated blade caused difficulty in advancing the tracheal tube to the glottic entrance, because both pharynx and the glottis were not under direct view, resulting in a partly blind oropharyngeal passage of the styletted tube. For avoidance of such complications, insertion and oropharyngeal passage of the endotracheal tube should be directly visualised as long as possible and training on the device combined with a good technique is mandatory. Further, this may result into prolonged intubation time [25]. In contrast, due to the lower angle of the C-MAC blade, the tip of the blade may always be seen on the video screen; the association between blade angulations and both visualisation and intubation success has been addressed by a recent article of Levitan et al. [26]. Compared to highly-angulated blades that provide optimal glottic visualisation (C/L 1) in most cases, but sometimes at the expense of more difficult tube advancement and subsequent intubation success, glottic exposure with the C-MAC may be incomplete (C/L 2a+b) in a higher proportion of cases but may allow easier intubation conditions anyway. All participating medical personnel are enabled to follow both visualisation of the glottis and intubation process on the monitor, and may help optimising glottic view by external laryngeal manipulations, since manoeuvres such as BURP may improve glottic visualisation both with conventional and videolaryngoscopy, as shown in the present study.

Videolaryngoscopy is not a technique to make endotracheal intubation faster, as shown in the present study: Time to successful intubation was quite comparable between direct laryngoscopy and videolaryngoscopy. However, it may help to make intubation safer. First, as shown previously, video-assisted laryngoscopes reduce the applied forces to the maxillary incisors as an objective measurement of intubation difficulty over standard blades [17]. Second, compared to conventional Macintosh laryngoscopy, videolaryngoscopy, particularly with the C-MAC, has been shown in a manikin model to result in better visualisation, easier use, and faster intubation time [5]. In the present clinical study, we were able to show that if one encounters unexpected difficulty of direct laryngoscopy, the use of the C-MAC size 4 may be advantageous if it is combined with the "straight blade technique", directly elevating the visualised epiglottis with the tip of the blade. Using this technique, we were able to improve the C/L class and subsequently intubation in 15 of 24 patients with suboptimal glottic visualisation.

As expected, subjective handling in patients with good or acceptable glottic view was best with the conventional Macintosh laryngoscope, which may result from the greater familiarity with this device (handle, grip, etc.); however, there were no differences between devices, or even a slight advantage for C-MAC3 and C-MAC4, if glottic visualisation was poor.

Some limitations of this study should be noted. First, we have included seven fasting patients with morbid obesity. It may be criticised that these patients may have a higher risk of regurgitation and aspiration of gastric content; however, standard institutional anaesthetic management for obese patients was applied, and all patients were successfully managed without complications. Second, fogging of the optical lens was transiently observed in 11 of 112 patients. As a result, the manufacturer has optimised the pre-heating system of the lens; thereafter, we did not observe any case of fogging in the remaining 38 patients. However, since fogging occurred transiently, it had no impact on intubation success. In two cases, dazzling (e.g. reflexions or inadequate luminance in comparison to bright surrounding light) of the monitor occurred, which is a common problem with videolaryngoscopy; however, both fogging and dazzling only may be deleterious in an emergency airway situation, if direct laryngoscopic view would not be possible. Third, our intraoperative data collection was performed by a non-blinded observer, which is possible source of bias. Finally, both data of ease or difficulty of intubation and handling the airway devices were subjective.

Footnote: ^a ^In the meanwhile, both GlideScope and McGrath videolaryngoscopes have been presented with Macintosh blade shapes.

## Conclusions

Combining the benefits of conventional direct laryngoscopy and videolaryngoscopy in one device, the C-MAC may serve as a standard intubation device for both routine airway management and educational purposes. In patients with impeded glottic view (C/L≥2a), the C-MAC size 4 with straight blade technique may reduce the number of C/L 3 or C/L 4 views, and therefore facilitate intubation. Further studies on patients with difficult airway should be performed to confirm these findings.

## Competing interests

Funding was restricted to institutional and departmental sources. The University Hospital Schleswig-Holstein Campus Kiel, Department of Anaesthesiology and Intensive Care Medicine, or any of its employees, receive no compensation for this work. However, Volker Doerges is a member of the Karl Storz advisory board, and receives grant support from Karl Storz, Tuttlingen, Germany, for studies related to airway management.

## Authors' contributions

EC performed anaesthesia and (video-) laryngoscopy, analysis of the data and drafted the manuscript. CT performed data acquisition and data entry into computer software. TM performed anaesthesia and (video-) laryngoscopy and had contribution on drafting the manuscript (Methods section). JK performed anaesthesia and (video-) laryngoscopy and helped in data acquisition. VD conceived of the study and helped to draft the manuscript (Discussion section). KW participated in the study design and helped to draft the manuscript. All authors read and approved the final manuscript.

## Pre-publication history

The pre-publication history for this paper can be accessed here:

http://www.biomedcentral.com/1471-2253/11/6/prepub
